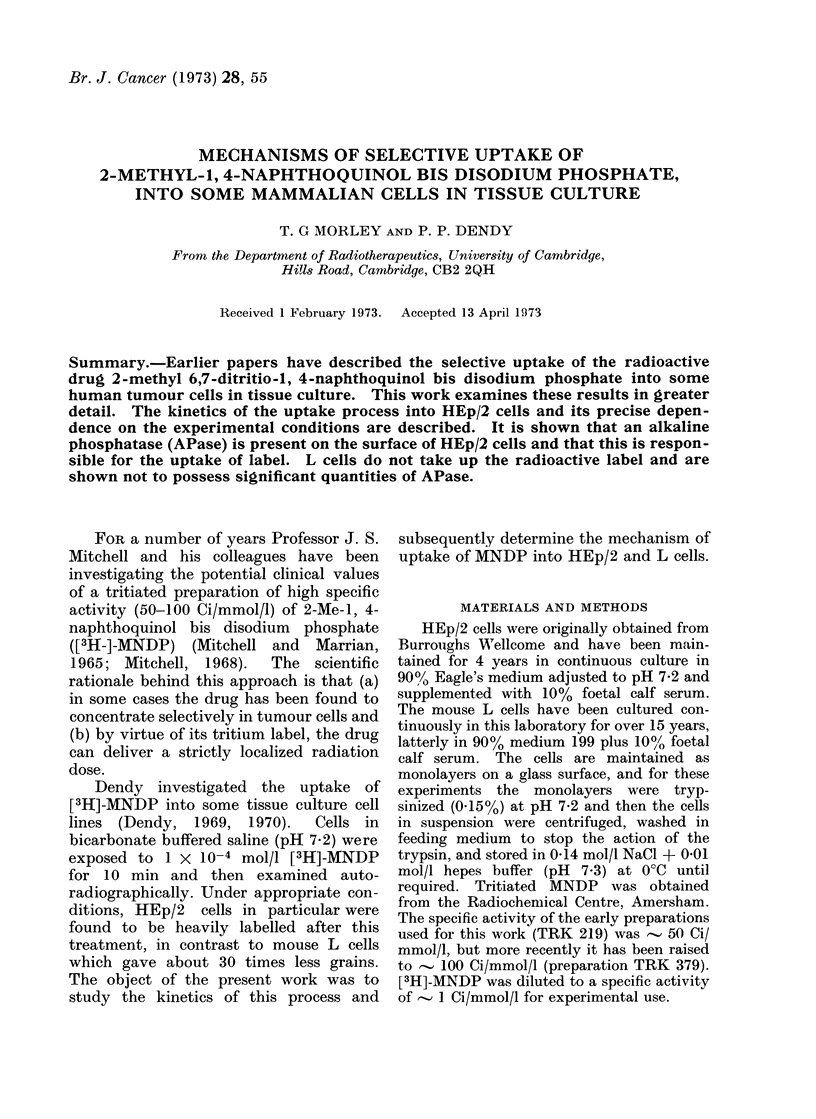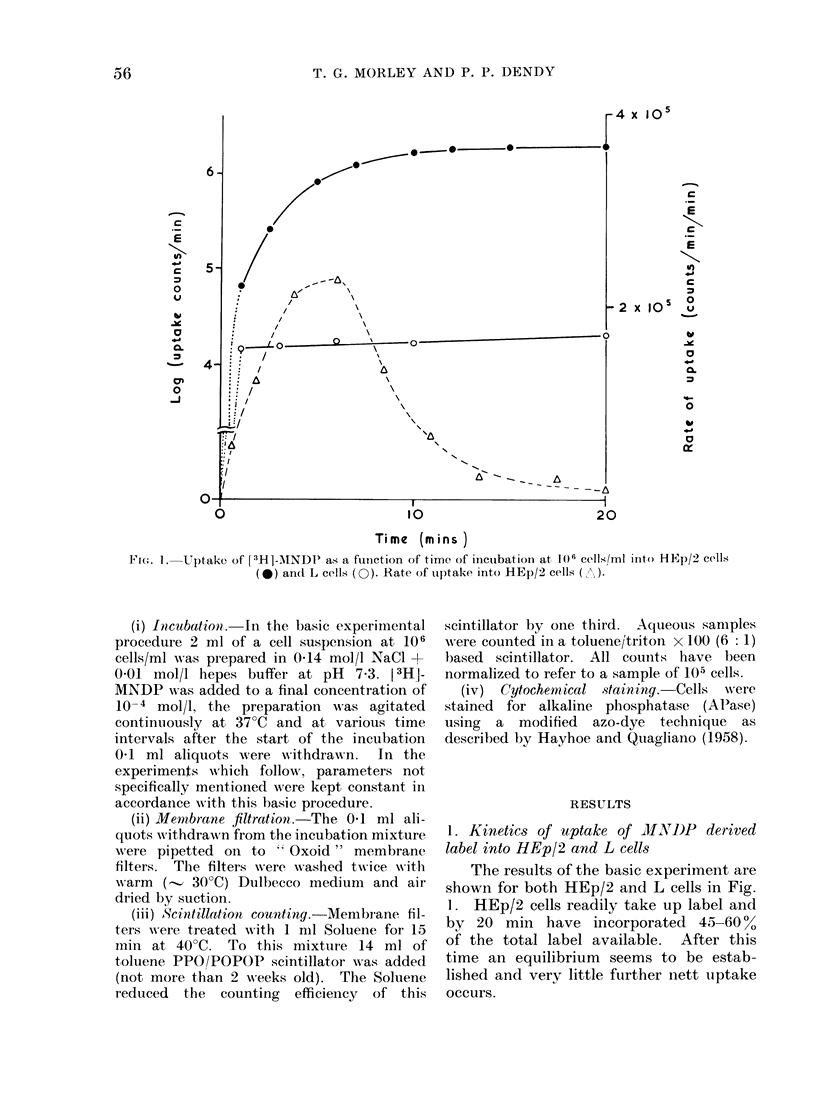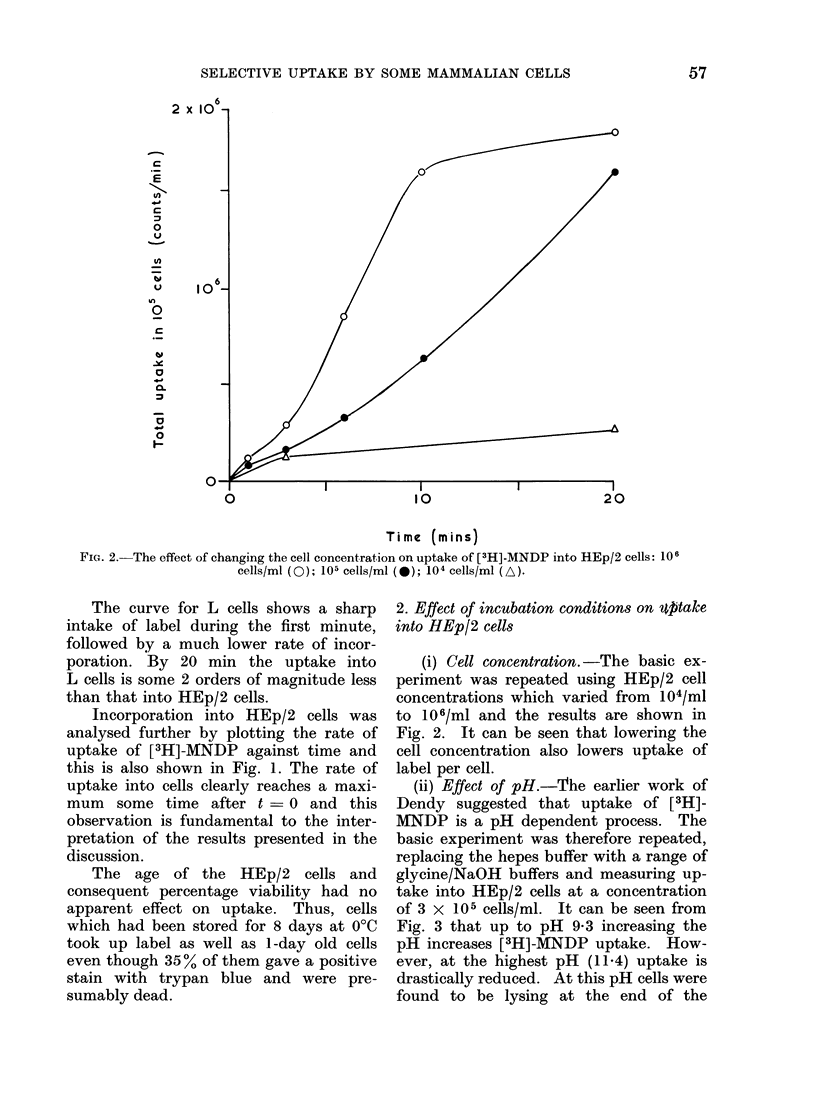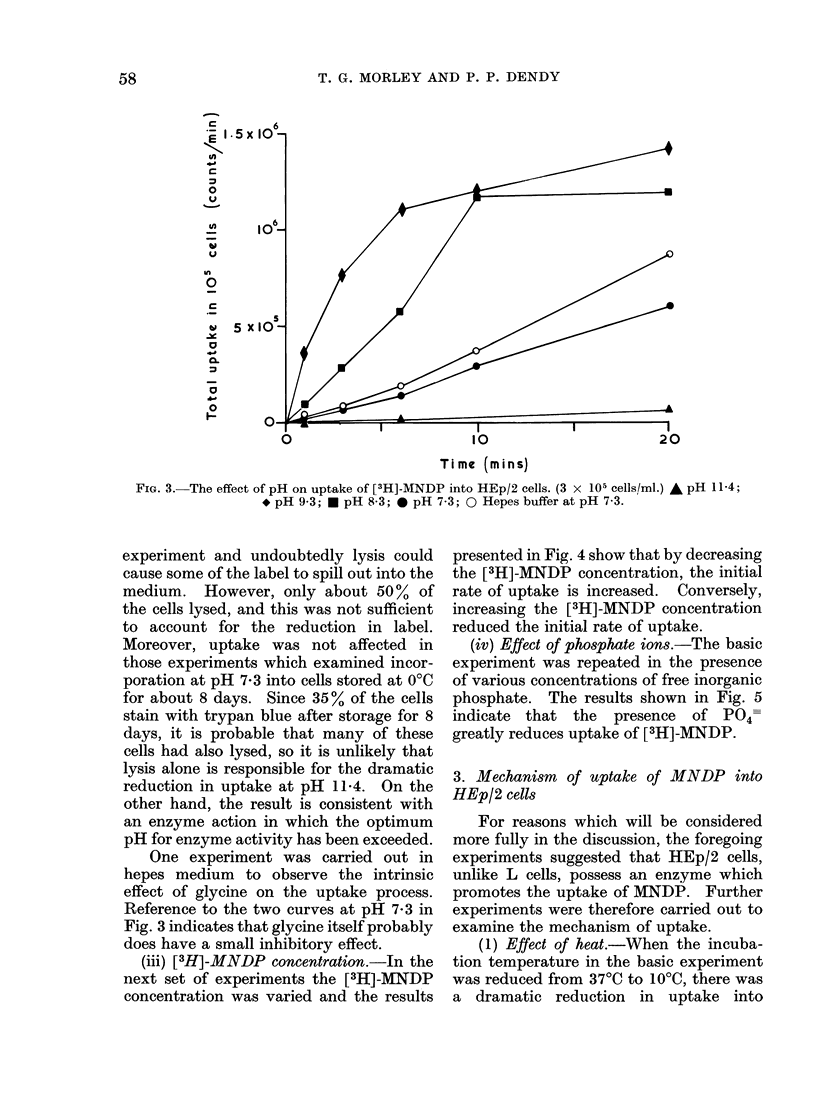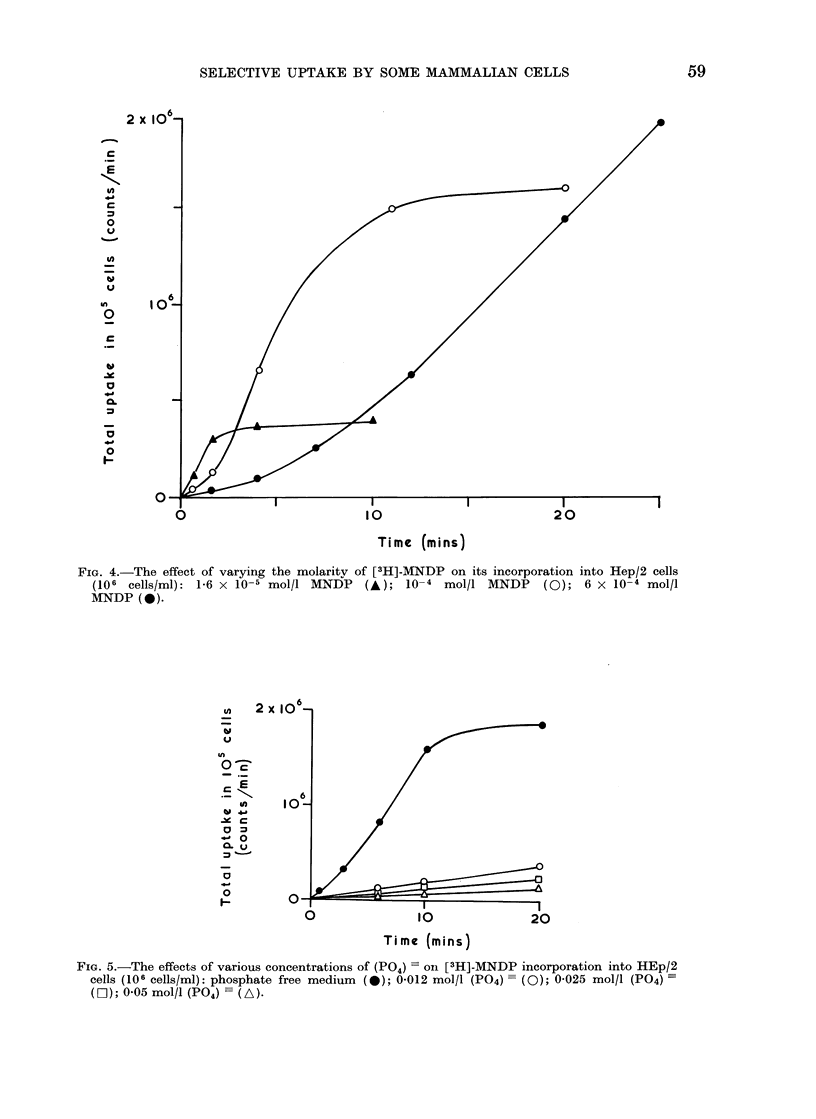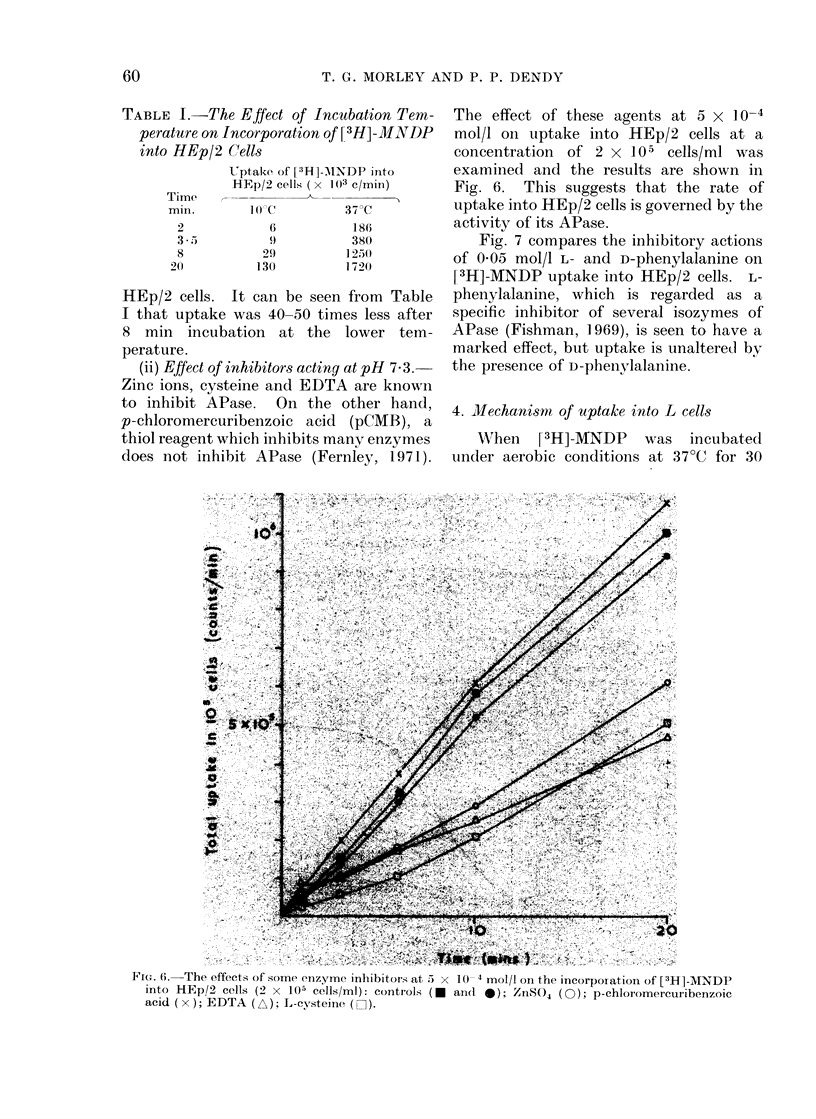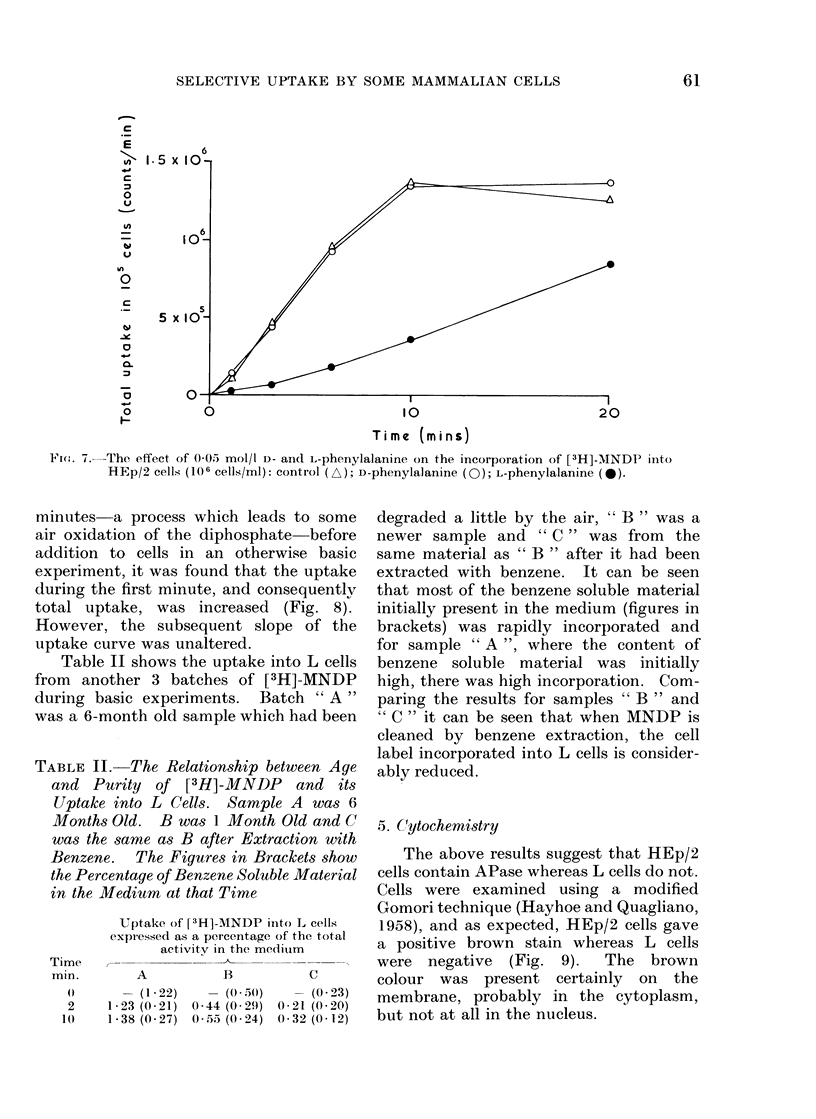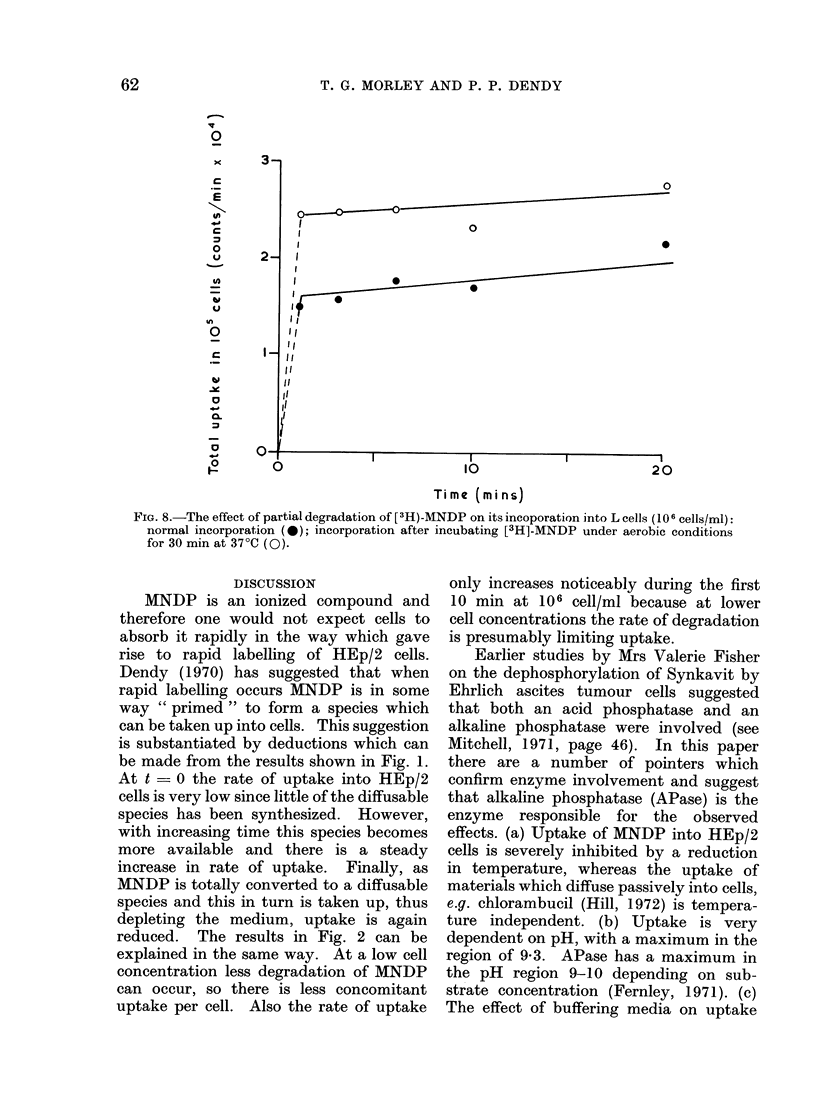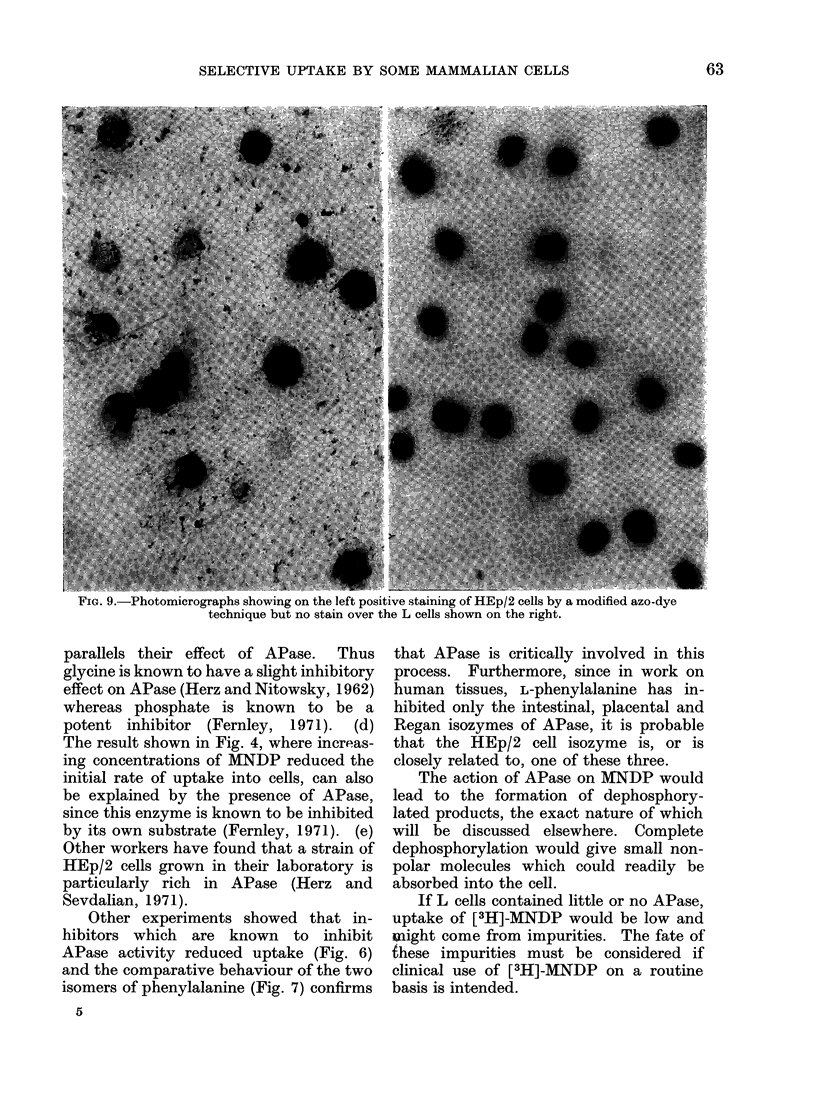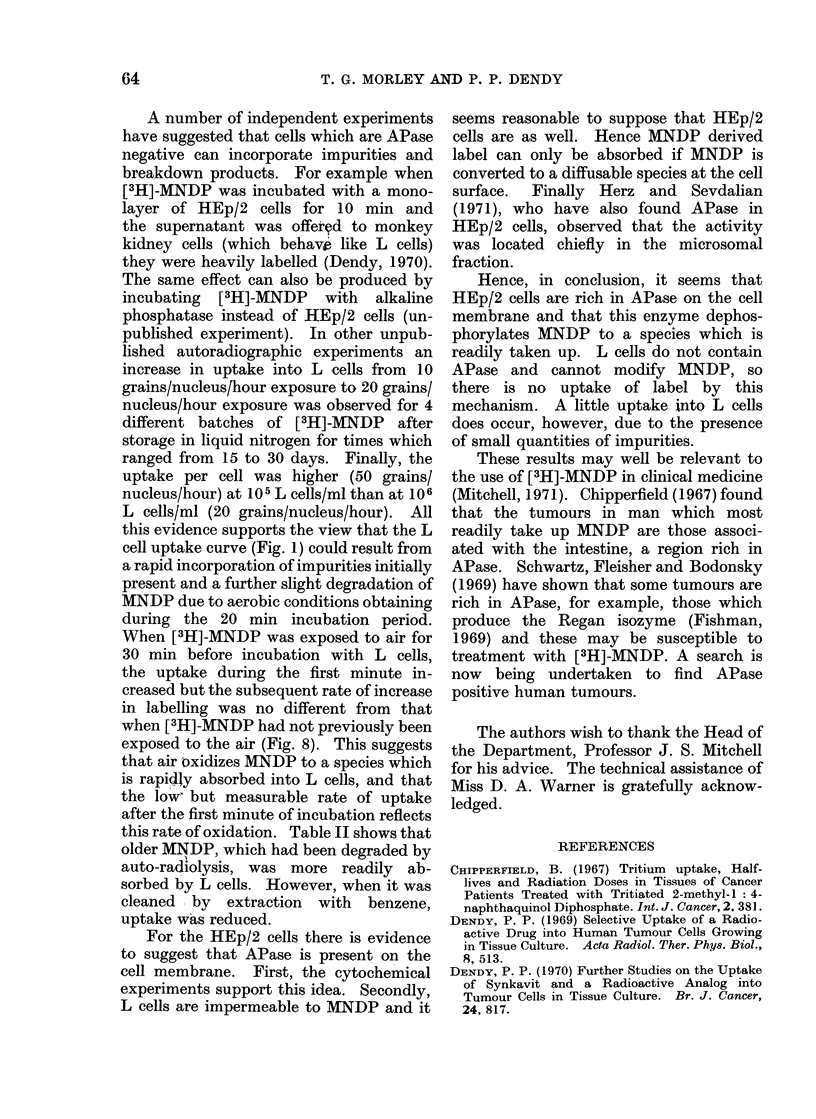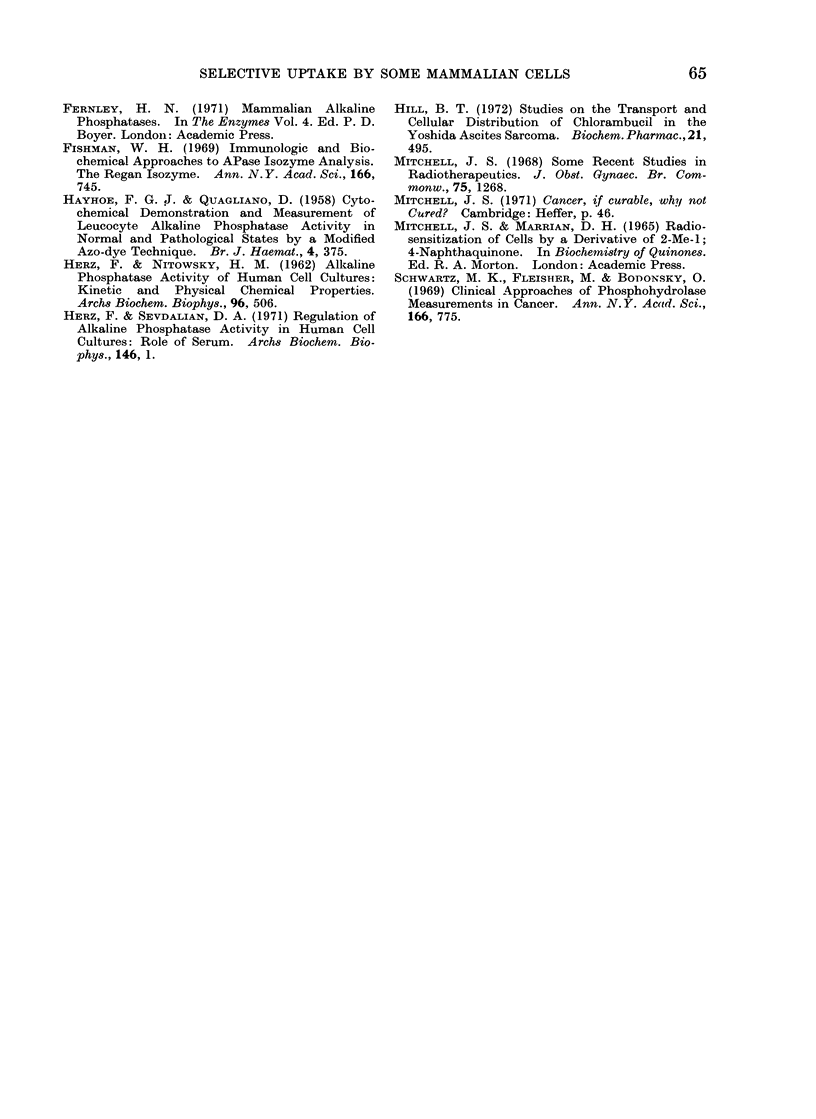# Mechanisms of Selective Uptake of 2-Methyl-1, 4-Naphthoquinol Bis Disodium Phosphate, into some Mammalian Cells in Tissue Culture

**DOI:** 10.1038/bjc.1973.70

**Published:** 1973-07

**Authors:** T. G Morley, P. P. Dendy

## Abstract

**Images:**


*Br. J. Cancer* (1973) **28**, 55

### MECHANISMS OF SELECTIVE UPTAKE OF 2-METHYL-1, 4-NAPHTHOQUINOL BIS DISODIUM PHOSPHATE, INTO SOME MAMMALIAN CELLS IN TISSUE CULTURE

T. G MORLEY AND P. P. DENDY

*From the Department of Radiotherapeutics, University of Cambridge, Hills Road, Cambridge*, CB2 2QH

Received 1 February 1973. Accepted 13 April 1973

**Summary.—Earlier papers have described the selective uptake of the radioactive drug 2-methyl 6,7-ditritio-1, 4-naphthoquinol bis disodium phosphate into some human tumour cells in tissue culture. This work examines these results in greater detail. The kinetics of the uptake process into HEp/2 cells and its precise dependence on the experimental conditions are described. It is shown that an alkaline phosphatase (APase) is present on the surface of HEp/2 cells and that this is responsible for the uptake of label. L cells do not take up the radioactive label and are shown not to possess significant quantities of APase.**

For a number of years Professor J. S. Mitchell and his colleagues have been investigating the potential clinical values of a tritiated preparation of high specific activity (50–100 Ci/mmol/l) of 2-Me-1, 4-naphthoquinol bis disodium phosphate ([$^3$H-]-MNDP) (Mitchell and Marrian, 1965; Mitchell, 1968). The scientific rationale behind this approach is that (a) in some cases the drug has been found to concentrate selectively in tumour cells and (b) by virtue of its tritium label, the drug can deliver a strictly localized radiation dose.

Dendy investigated the uptake of [$^3$H]-MNDP into some tissue culture cell lines (Dendy, 1969, 1970). Cells in bicarbonate buffered saline (pH 7·2) were exposed to $1 \times 10^{-4}$ mol/l [$^3$H]-MNDP for 10 min and then examined autoradiographically. Under appropriate conditions, HEp/2 cells in particular were found to be heavily labelled after this treatment, in contrast to mouse L cells which gave about 30 times less grains. The object of the present work was to study the kinetics of this process and subsequently determine the mechanism of uptake of MNDP into HEp/2 and L cells.

### MATERIALS AND METHODS

HEp/2 cells were originally obtained from Burroughs Wellcome and have been maintained for 4 years in continuous culture in 90% Eagle's medium adjusted to pH 7·2 and supplemented with 10% foetal calf serum. The mouse L cells have been cultured continuously in this laboratory for over 15 years, latterly in 90% medium 199 plus 10% foetal calf serum. The cells are maintained as monolayers on a glass surface, and for these experiments the monolayers were trypsinized (0·15%) at pH 7·2 and then the cells in suspension were centrifuged, washed in feeding medium to stop the action of the trypsin, and stored in 0·14 mol/l NaCl + 0·01 mol/l hepes buffer (pH 7·3) at 0°C until required. Tritiated MNDP was obtained from the Radiochemical Centre, Amersham. The specific activity of the early preparations used for this work (TRK 219) was ~ 50 Ci/mmol/l, but more recently it has been raised to ~ 100 Ci/mmol/l (preparation TRK 379). [$^3$H]-MNDP was diluted to a specific activity of ~ 1 Ci/mmol/l for experimental use.

FIG. 1.—Uptake of [$^3$H]-MNDP as a function of time of incubation at $10^6$ cells/ml into HEp/2 cells (●) and L cells (○). Rate of uptake into HEp/2 cells (△).

(i) *Incubation.*—In the basic experimental procedure 2 ml of a cell suspension at $10^6$ cells/ml was prepared in 0·14 mol/l NaCl + 0·01 mol/l hepes buffer at pH 7·3. [$^3$H]-MNDP was added to a final concentration of $10^{-4}$ mol/l, the preparation was agitated continuously at 37°C and at various time intervals after the start of the incubation 0·1 ml aliquots were withdrawn. In the experiments which follow, parameters not specifically mentioned were kept constant in accordance with this basic procedure.

(ii) *Membrane filtration.*—The 0·1 ml aliquots withdrawn from the incubation mixture were pipetted on to "Oxoid" membrane filters. The filters were washed twice with warm ($\sim$ 30°C) Dulbecco medium and air dried by suction.

(iii) *Scintillation counting.*—Membrane filters were treated with 1 ml Soluene for 15 min at 40°C. To this mixture 14 ml of toluene PPO/POPOP scintillator was added (not more than 2 weeks old). The Soluene reduced the counting efficiency of this scintillator by one third. Aqueous samples were counted in a toluene/triton × 100 (6 : 1) based scintillator. All counts have been normalized to refer to a sample of $10^5$ cells.

(iv) *Cytochemical staining.*—Cells were stained for alkaline phosphatase (APase) using a modified azo-dye technique as described by Hayhoe and Quagliano (1958).

### RESULTS

### 1. *Kinetics of uptake of MNDP derived label into HEp/2 and L cells*

The results of the basic experiment are shown for both HEp/2 and L cells in Fig. 1. HEp/2 cells readily take up label and by 20 min have incorporated 45–60% of the total label available. After this time an equilibrium seems to be established and very little further nett uptake occurs.

FIG. 2.—The effect of changing the cell concentration on uptake of [$^3$H]-MNDP into HEp/2 cells: $10^6$ cells/ml (○); $10^5$ cells/ml (●); $10^4$ cells/ml (△).

The curve for L cells shows a sharp intake of label during the first minute, followed by a much lower rate of incorporation. By 20 min the uptake into L cells is some 2 orders of magnitude less than that into HEp/2 cells.

Incorporation into HEp/2 cells was analysed further by plotting the rate of uptake of [$^3$H]-MNDP against time and this is also shown in Fig. 1. The rate of uptake into cells clearly reaches a maximum some time after $t = 0$ and this observation is fundamental to the interpretation of the results presented in the discussion.

The age of the HEp/2 cells and consequent percentage viability had no apparent effect on uptake. Thus, cells which had been stored for 8 days at 0°C took up label as well as 1-day old cells even though 35% of them gave a positive stain with trypan blue and were presumably dead.

2. *Effect of incubation conditions on uptake into HEp/2 cells*

(i) *Cell concentration.*—The basic experiment was repeated using HEp/2 cell concentrations which varied from $10^4$/ml to $10^6$/ml and the results are shown in Fig. 2. It can be seen that lowering the cell concentration also lowers uptake of label per cell.

(ii) *Effect of pH.*—The earlier work of Dendy suggested that uptake of [$^3$H]-MNDP is a pH dependent process. The basic experiment was therefore repeated, replacing the hepes buffer with a range of glycine/NaOH buffers and measuring uptake into HEp/2 cells at a concentration of $3 \times 10^5$ cells/ml. It can be seen from Fig. 3 that up to pH 9·3 increasing the pH increases [$^3$H]-MNDP uptake. However, at the highest pH (11·4) uptake is drastically reduced. At this pH cells were found to be lysing at the end of the

FIG. 3.—The effect of pH on uptake of [$^3$H]-MNDP into HEp/2 cells. (3 × $10^5$ cells/ml.) ▲ pH 11·4; ◆ pH 9·3; ■ pH 8·3; ● pH 7·3; ○ Hepes buffer at pH 7·3.

experiment and undoubtedly lysis could cause some of the label to spill out into the medium. However, only about 50% of the cells lysed, and this was not sufficient to account for the reduction in label. Moreover, uptake was not affected in those experiments which examined incorporation at pH 7·3 into cells stored at 0°C for about 8 days. Since 35% of the cells stain with trypan blue after storage for 8 days, it is probable that many of these cells had also lysed, so it is unlikely that lysis alone is responsible for the dramatic reduction in uptake at pH 11·4. On the other hand, the result is consistent with an enzyme action in which the optimum pH for enzyme activity has been exceeded.

One experiment was carried out in hepes medium to observe the intrinsic effect of glycine on the uptake process. Reference to the two curves at pH 7·3 in Fig. 3 indicates that glycine itself probably does have a small inhibitory effect.

(iii) *[$^3$H]-MNDP concentration*.—In the next set of experiments the [$^3$H]-MNDP concentration was varied and the results presented in Fig. 4 show that by decreasing the [$^3$H]-MNDP concentration, the initial rate of uptake is increased. Conversely, increasing the [$^3$H]-MNDP concentration reduced the initial rate of uptake.

(*iv*) *Effect of phosphate ions*.—The basic experiment was repeated in the presence of various concentrations of free inorganic phosphate. The results shown in Fig. 5 indicate that the presence of $PO_4^{\equiv}$ greatly reduces uptake of [$^3$H]-MNDP.

### 3. *Mechanism of uptake of MNDP into HEp/2 cells*

For reasons which will be considered more fully in the discussion, the foregoing experiments suggested that HEp/2 cells, unlike L cells, possess an enzyme which promotes the uptake of MNDP. Further experiments were therefore carried out to examine the mechanism of uptake.

(1) *Effect of heat*.—When the incubation temperature in the basic experiment was reduced from 37°C to 10°C, there was a dramatic reduction in uptake into

FIG. 4.—The effect of varying the molarity of [$^3$H]-MNDP on its incorporation into Hep/2 cells ($10^6$ cells/ml): $1{\cdot}6 \times 10^{-5}$ mol/l MNDP (▲); $10^{-4}$ mol/l MNDP (○); $6 \times 10^{-4}$ mol/l MNDP (●).

FIG. 5.—The effects of various concentrations of $(PO_4)^{\equiv}$ on [$^3$H]-MNDP incorporation into HEp/2 cells ($10^6$ cells/ml): phosphate free medium (●); 0·012 mol/l $(PO_4)^{\equiv}$ (○); 0·025 mol/l $(PO_4)^{\equiv}$ (□); 0·05 mol/l $(PO_4)^{\equiv}$ (△).

TABLE I.—*The Effect of Incubation Temperature on Incorporation of [³H]-MNDP into HEp/2 Cells*

| Time min. | Uptake of [³H]-MNDP into HEp/2 cells (× $10^3$ c/min) 10°C | 37°C |
|---|---|---|
| 2 | 6 | 186 |
| 3·5 | 9 | 380 |
| 8 | 29 | 1250 |
| 20 | 130 | 1720 |

HEp/2 cells. It can be seen from Table I that uptake was 40–50 times less after 8 min incubation at the lower temperature.

(ii) *Effect of inhibitors acting at pH* 7·3.—Zinc ions, cysteine and EDTA are known to inhibit APase. On the other hand, *p*-chloromercuribenzoic acid (pCMB), a thiol reagent which inhibits many enzymes does not inhibit APase (Fernley, 1971). The effect of these agents at $5 \times 10^{-4}$ mol/l on uptake into HEp/2 cells at a concentration of $2 \times 10^5$ cells/ml was examined and the results are shown in Fig. 6. This suggests that the rate of uptake into HEp/2 cells is governed by the activity of its APase.

Fig. 7 compares the inhibitory actions of 0·05 mol/l L- and D-phenylalanine on [³H]-MNDP uptake into HEp/2 cells. L-phenylalanine, which is regarded as a specific inhibitor of several isozymes of APase (Fishman, 1969), is seen to have a marked effect, but uptake is unaltered by the presence of D-phenylalanine.

4. *Mechanism of uptake into L cells*

When [³H]-MNDP was incubated under aerobic conditions at 37°C for 30

FIG. 6.—The effects of some enzyme inhibitors at $5 \times 10^{-4}$ mol/l on the incorporation of [³H]-MNDP into HEp/2 cells ($2 \times 10^5$ cells/ml): controls (■ and ●); $ZnSO_4$ (○); p-chloromercuribenzoic acid (×); EDTA (△); L-cysteine (□).

FIG. 7.—The effect of 0·05 mol/l D- and L-phenylalanine on the incorporation of [$^3$H]-MNDP into HEp/2 cells ($10^6$ cells/ml): control (△); D-phenylalanine (○); L-phenylalanine (●).

minutes—a process which leads to some air oxidation of the diphosphate—before addition to cells in an otherwise basic experiment, it was found that the uptake during the first minute, and consequently total uptake, was increased (Fig. 8). However, the subsequent slope of the uptake curve was unaltered.

Table II shows the uptake into L cells from another 3 batches of [$^3$H]-MNDP during basic experiments. Batch " A " was a 6-month old sample which had been degraded a little by the air, " B " was a newer sample and " C " was from the same material as " B " after it had been extracted with benzene. It can be seen that most of the benzene soluble material initially present in the medium (figures in brackets) was rapidly incorporated and for sample " A ", where the content of benzene soluble material was initially high, there was high incorporation. Comparing the results for samples " B " and " C " it can be seen that when MNDP is cleaned by benzene extraction, the cell label incorporated into L cells is considerably reduced.

TABLE II.—*The Relationship between Age and Purity of [$^3$H]-MNDP and its Uptake into L Cells. Sample A was 6 Months Old. B was 1 Month Old and C was the same as B after Extraction with Benzene. The Figures in Brackets show the Percentage of Benzene Soluble Material in the Medium at that Time*

| Time min. | Uptake of [$^3$H]-MNDP into L cells expressed as a percentage of the total activity in the medium A | B | C |
|---|---|---|---|
| 0 | — (1·22) | — (0·50) | — (0·23) |
| 2 | 1·23 (0·21) | 0·44 (0·29) | 0·21 (0·20) |
| 10 | 1·38 (0·27) | 0·55 (0·24) | 0·32 (0·12) |

5. *Cytochemistry*

The above results suggest that HEp/2 cells contain APase whereas L cells do not. Cells were examined using a modified Gomori technique (Hayhoe and Quagliano, 1958), and as expected, HEp/2 cells gave a positive brown stain whereas L cells were negative (Fig. 9). The brown colour was present certainly on the membrane, probably in the cytoplasm, but not at all in the nucleus.

FIG. 8.—The effect of partial degradation of [$^3$H)-MNDP on its incoporation into L cells ($10^6$ cells/ml): normal incorporation (●); incorporation after incubating [$^3$H]-MNDP under aerobic conditions for 30 min at 37°C (○).

DISCUSSION

MNDP is an ionized compound and therefore one would not expect cells to absorb it rapidly in the way which gave rise to rapid labelling of HEp/2 cells. Dendy (1970) has suggested that when rapid labelling occurs MNDP is in some way " primed " to form a species which can be taken up into cells. This suggestion is substantiated by deductions which can be made from the results shown in Fig. 1. At $t = 0$ the rate of uptake into HEp/2 cells is very low since little of the diffusable species has been synthesized. However, with increasing time this species becomes more available and there is a steady increase in rate of uptake. Finally, as MNDP is totally converted to a diffusable species and this in turn is taken up, thus depleting the medium, uptake is again reduced. The results in Fig. 2 can be explained in the same way. At a low cell concentration less degradation of MNDP can occur, so there is less concomitant uptake per cell. Also the rate of uptake only increases noticeably during the first 10 min at $10^6$ cell/ml because at lower cell concentrations the rate of degradation is presumably limiting uptake.

Earlier studies by Mrs Valerie Fisher on the dephosphorylation of Synkavit by Ehrlich ascites tumour cells suggested that both an acid phosphatase and an alkaline phosphatase were involved (see Mitchell, 1971, page 46). In this paper there are a number of pointers which confirm enzyme involvement and suggest that alkaline phosphatase (APase) is the enzyme responsible for the observed effects. (a) Uptake of MNDP into HEp/2 cells is severely inhibited by a reduction in temperature, whereas the uptake of materials which diffuse passively into cells, *e.g.* chlorambucil (Hill, 1972) is temperature independent. (b) Uptake is very dependent on pH, with a maximum in the region of 9·3. APase has a maximum in the pH region 9–10 depending on substrate concentration (Fernley, 1971). (c) The effect of buffering media on uptake

FIG. 9.—Photomicrographs showing on the left positive staining of HEp/2 cells by a modified azo-dye technique but no stain over the L cells shown on the right.

parallels their effect of APase. Thus glycine is known to have a slight inhibitory effect on APase (Herz and Nitowsky, 1962) whereas phosphate is known to be a potent inhibitor (Fernley, 1971). (d) The result shown in Fig. 4, where increasing concentrations of MNDP reduced the initial rate of uptake into cells, can also be explained by the presence of APase, since this enzyme is known to be inhibited by its own substrate (Fernley, 1971). (e) Other workers have found that a strain of HEp/2 cells grown in their laboratory is particularly rich in APase (Herz and Sevdalian, 1971).

Other experiments showed that inhibitors which are known to inhibit APase activity reduced uptake (Fig. 6) and the comparative behaviour of the two isomers of phenylalanine (Fig. 7) confirms that APase is critically involved in this process. Furthermore, since in work on human tissues, L-phenylalanine has inhibited only the intestinal, placental and Regan isozymes of APase, it is probable that the HEp/2 cell isozyme is, or is closely related to, one of these three.

The action of APase on MNDP would lead to the formation of dephosphorylated products, the exact nature of which will be discussed elsewhere. Complete dephosphorylation would give small nonpolar molecules which could readily be absorbed into the cell.

If L cells contained little or no APase, uptake of [$^3$H]-MNDP would be low and might come from impurities. The fate of these impurities must be considered if clinical use of [$^3$H]-MNDP on a routine basis is intended.

A number of independent experiments have suggested that cells which are APase negative can incorporate impurities and breakdown products. For example when [$^3$H]-MNDP was incubated with a monolayer of HEp/2 cells for 10 min and the supernatant was offered to monkey kidney cells (which behave like L cells) they were heavily labelled (Dendy, 1970). The same effect can also be produced by incubating [$^3$H]-MNDP with alkaline phosphatase instead of HEp/2 cells (unpublished experiment). In other unpublished autoradiographic experiments an increase in uptake into L cells from 10 grains/nucleus/hour exposure to 20 grains/nucleus/hour exposure was observed for 4 different batches of [$^3$H]-MNDP after storage in liquid nitrogen for times which ranged from 15 to 30 days. Finally, the uptake per cell was higher (50 grains/nucleus/hour) at $10^5$ L cells/ml than at $10^6$ L cells/ml (20 grains/nucleus/hour). All this evidence supports the view that the L cell uptake curve (Fig. 1) could result from a rapid incorporation of impurities initially present and a further slight degradation of MNDP due to aerobic conditions obtaining during the 20 min incubation period. When [$^3$H]-MNDP was exposed to air for 30 min before incubation with L cells, the uptake during the first minute increased but the subsequent rate of increase in labelling was no different from that when [$^3$H]-MNDP had not previously been exposed to the air (Fig. 8). This suggests that air oxidizes MNDP to a species which is rapidly absorbed into L cells, and that the low but measurable rate of uptake after the first minute of incubation reflects this rate of oxidation. Table II shows that older MNDP, which had been degraded by auto-radiolysis, was more readily absorbed by L cells. However, when it was cleaned by extraction with benzene, uptake was reduced.

For the HEp/2 cells there is evidence to suggest that APase is present on the cell membrane. First, the cytochemical experiments support this idea. Secondly, L cells are impermeable to MNDP and it seems reasonable to suppose that HEp/2 cells are as well. Hence MNDP derived label can only be absorbed if MNDP is converted to a diffusable species at the cell surface. Finally Herz and Sevdalian (1971), who have also found APase in HEp/2 cells, observed that the activity was located chiefly in the microsomal fraction.

Hence, in conclusion, it seems that HEp/2 cells are rich in APase on the cell membrane and that this enzyme dephosphorylates MNDP to a species which is readily taken up. L cells do not contain APase and cannot modify MNDP, so there is no uptake of label by this mechanism. A little uptake into L cells does occur, however, due to the presence of small quantities of impurities.

These results may well be relevant to the use of [$^3$H]-MNDP in clinical medicine (Mitchell, 1971). Chipperfield (1967) found that the tumours in man which most readily take up MNDP are those associated with the intestine, a region rich in APase. Schwartz, Fleisher and Bodonsky (1969) have shown that some tumours are rich in APase, for example, those which produce the Regan isozyme (Fishman, 1969) and these may be susceptible to treatment with [$^3$H]-MNDP. A search is now being undertaken to find APase positive human tumours.

The authors wish to thank the Head of the Department, Professor J. S. Mitchell for his advice. The technical assistance of Miss D. A. Warner is gratefully acknowledged.

### REFERENCES

Chipperfield, B. (1967) Tritium uptake, Half-lives and Radiation Doses in Tissues of Cancer Patients Treated with Tritiated 2-methyl-1 : 4-naphthaquinol Diphosphate. *Int. J. Cancer*, **2**, 381.

Dendy, P. P. (1969) Selective Uptake of a Radioactive Drug into Human Tumour Cells Growing in Tissue Culture. *Acta Radiol. Ther. Phys. Biol.*, **8**, 513.

Dendy, P. P. (1970) Further Studies on the Uptake of Synkavit and a Radioactive Analog into Tumour Cells in Tissue Culture. *Br. J. Cancer*, **24**, 817.

FERNLEY, H. N. (1971) Mammalian Alkaline Phosphatases. In *The Enzymes* Vol. 4. Ed. P. D. Boyer. London: Academic Press.

FISHMAN, W. H. (1969) Immunologic and Biochemical Approaches to APase Isozyme Analysis. The Regan Isozyme. *Ann. N.Y. Acad. Sci.*, **166**, 745.

HAYHOE, F. G. J. & QUAGLIANO, D. (1958) Cytochemical Demonstration and Measurement of Leucocyte Alkaline Phosphatase Activity in Normal and Pathological States by a Modified Azo-dye Technique. *Br. J. Haemat.*, **4**, 375.

HERZ, F. & NITOWSKY, H. M. (1962) Alkaline Phosphatase Activity of Human Cell Cultures: Kinetic and Physical Chemical Properties. *Archs Biochem. Biophys.*, **96**, 506.

HERZ, F. & SEVDALIAN, D. A. (1971) Regulation of Alkaline Phosphatase Activity in Human Cell Cultures: Role of Serum. *Archs Biochem. Biophys.*, **146**, 1.

HILL, B. T. (1972) Studies on the Transport and Cellular Distribution of Chlorambucil in the Yoshida Ascites Sarcoma. *Biochem. Pharmac.*, **21**, 495.

MITCHELL, J. S. (1968) Some Recent Studies in Radiotherapeutics. *J. Obst. Gynaec. Br. Commonw.*, **75**, 1268.

MITCHELL, J. S. (1971) *Cancer, if curable, why not Cured?* Cambridge: Heffer, p. 46.

MITCHELL, J. S. & MARRIAN, D. H. (1965) Radiosensitization of Cells by a Derivative of 2-Me-1; 4-Naphthaquinone. In *Biochemistry of Quinones*. Ed. R. A. Morton. London: Academic Press.

SCHWARTZ, M. K., FLEISHER, M. & BODONSKY, O. (1969) Clinical Approaches of Phosphohydrolase Measurements in Cancer. *Ann. N.Y. Acad. Sci.*, **166**, 775.